# Accuracy of Gene Expression Prediction From Genotype Data With PrediXcan Varies Across and Within Continental Populations

**DOI:** 10.3389/fgene.2019.00261

**Published:** 2019-04-03

**Authors:** Anna V. Mikhaylova, Timothy A. Thornton

**Affiliations:** Department of Biostatistics, University of Washington, Seattle, WA, United States

**Keywords:** transcriptome, expression quantitative trait loci (eQTL), genetic diversity, genetic mapping, complex traits

## Abstract

Using genetic data to predict gene expression has garnered significant attention in recent years. PrediXcan has become one of the most widely used gene-based methods for testing associations between predicted gene expression values and a phenotype, which has facilitated novel insights into the relationship between complex traits and the component of gene expression that can be attributed to genetic variation. The gene expression prediction models for PrediXcan were developed using supervised machine learning methods and training data from the Depression Genes and Networks (DGN) study and the Genotype-Tissue Expression (GTEx) project, where the majority of subjects are of European descent. Many genetic studies, however, include samples from multi-ethnic populations, and in this paper we evaluate the accuracy of PrediXcan for predicting gene expression in diverse populations. Using transcriptomic data from the GEUVADIS (Genetic European Variation in Disease) RNA sequencing project and whole genome sequencing data from the 1000 Genomes project, we evaluate and compare the predictive performance of PrediXcan in an African population (Yoruban) and four European ancestry populations for thousands of genes. We evaluate a range of models from the PrediXcan weight databases and use Pearson's correlation coefficient to assess gene expression prediction accuracy with PrediXcan. From our evaluation, we find that the predictive performance of PrediXcan varies substantially among populations from different continents (*F*-test *p*-value < 2.2 × 10^−16^), where prediction accuracy is lower in the Yoruban population from West Africa compared to the European-ancestry populations. Moreover, not only do we find differences in predictive performance between populations from different continents, we also find highly significant differences in prediction accuracy among the four European ancestry populations considered (*F*-test *p*-value < 2.2 × 10^−16^). Finally, while there is variability in prediction accuracy across different PrediXcan weight databases, we also find consistency in the qualitative performance of PrediXcan for the five populations considered, with the African ancestry population having the lowest accuracy across databases.

## 1. Introduction

In the past decade, genome-wide association studies (GWAS) have identified thousands of genetic variants significantly associated with a wide range of human phenotypes (Sudlow et al., 2015; NHLBI, 2016; MacArthur et al., 2017; Visscher et al., 2017). The vast majority of these studies, however, were conducted in samples from European ancestry populations (Need and Goldstein, 2009; Bustamante et al., 2011; Petrovski and Goldstein, 2016; Popejoy and Fullerton, 2016; Bentley et al., 2017; Hindorff et al., 2018). Differences in allele frequencies, genetic architecture, and linkage disequilibrium (LD) patterns across ancestries suggest that GWAS discoveries can fail to generalize across populations, and recent publications have provided compelling evidence that GWAS findings often do not transfer from European populations to other ethnic groups (Adeyemo and Rotimi, 2009; Li and Keating, 2014). For example, Carlson et al. analyzed multi-ethnic data from the PAGE Consortium and concluded that some variants identified in GWAS in European ancestry populations had different magnitude and direction of allelic effects in non-European populations and the differential effects were more persistent in African Americans (Carlson et al., 2013). Moreover, genetic risk prediction models derived from European GWAS were found to be unreliable when applied to other ethnic groups (Carlson et al., 2013). Martin et al. examined the impact of population history on polygenic risk scores and demonstrated that they can be biased and confounded by population structure (Martin et al., 2017). Since genetic risk prediction accuracy depends on genetic similarity between the target and discovery cohorts, Martin et al. advised against interpreting the scores across populations and recommended computing them in genetically similar cohorts.

Associations between genetic variation and molecular traits, such as gene expression, have advanced our understanding of the mechanisms underlying trait-variant associations (Nica et al., 2010; Torres et al., 2014; Albert and Kruglyak, 2015). Prior studies have shown that a large proportion of GWAS variants identified for complex traits are expression quantitative trait loci (eQTLs), i.e., they play a role in regulating gene expression (Nicolae et al., 2010). Thus, eQTLs can aid in prioritizing likely causal variants among the ones identified by GWAS, especially if they are found in non-coding regions, and can help uncover the mechanisms by which genotypes influence phenotypes (Albert and Kruglyak, 2015). As a result, having three types of data— genotype, phenotype and gene expression—on the same set of subjects can be advantageous for improved understanding of the relationships between complex traits, the genetic backgrounds of study subjects, and the underlying biological processes. However, collecting all of these different types of data on the same study subjects is often not feasible due to cost and tissue availability. Additionally, eQTL studies have the same pitfalls as GWASs—the majority of the detected eQTLs are not causal, but may be in LD with causal variants. Similar to variants identified through GWAS, eQTL findings might fail to replicate in diverse populations due to differential LD patterns across populations (Kelly et al., 2017).

Recently, there has been increased interest in integrating eQTL studies and GWASs for improved complex trait mapping. PrediXcan (Gamazon et al., 2015) is one of the most widely used integrative methods for testing associations between a phenotype and gene expression values predicted from SNP genotyping or sequencing data. PrediXcan can have increased power over traditional GWAS methods, particularly when differential changes in gene expression is an intermediary stage of the causal pathway from genetic variation to the outcome of interest. A useful feature of PrediXcan (and other similar methods) is the ability to obtain predicted gene expression values on study subjects when tissue types relevant to phenotypes are not available. We now give a very brief overview of the PrediXcan method. PrediXcan uses machine learning methods and large reference datasets consisting of both genotype and trascriptome data for supervised training to construct prediction models for expression of each gene. With PrediXcan, genetic training data is restricted to common *cis*-variants that are within 1 Mb upstream and downstream from the transcription region (Gamazon et al., 2015). Gene-specific derived SNP weights from the prediction models are then stored in databases, with separate sets of weights for different tissue types. Using these weights, PrediXcan allows for the prediction of gene expression values for study subjects with available genotype data, where predicted expression values are computed as a weighted linear combination of SNP dosages. Finally, the predicted expression values can then be used to test for associations with a phenotype of interest. By conducting tests on gene expression obtained from an aggregation of variants, PrediXcan dramatically reduces multiple testing burden as compared to single variant association testing.

Previous studies have reported differences in gene expression levels across diverse populations from the HapMap3 project, noting that 77% of eQTLs are population specific and only 23% are shared between two or more populations (The International HapMap 3 Consortium et al., 2010; Stranger et al., 2012). More distantly related populations have more differentially expressed genes than closely related populations, although this can often be explained by the expression of different gene transcripts across populations (Lappalainen et al., 2013). One potential limitation of PrediXcan, however, is that the method may not perform well in diverse populations, as the supervised learning for PrediXcan was conducted using data from the Depression Genes and Networks (DGN) and the Genotype-Tissue Expression (GTEx) Project—both of which consist primarily of European-ancestry subjects (Lonsdale et al., 2013; Battle et al., 2014). Many genetic studies include samples from multi-ethnic populations, and understanding the accuracy of gene expression prediction with PrediXcan across populations is of interest to many genetic researchers.

Recent works have evaluated the performance of PrediXcan in diverse populations (Gottlieb et al., 2017; Li et al., 2018). Li et al. evaluated PrediXcan whole-blood prediction models and investigated the factors that influence prediction accuracy using the Yoruban (YRI) and European (CEU) samples from the Genetic European Variation in Health and Disease (GEUVADIS) (Lappalainen et al., 2013) cohort. In this paper, the PrediXcan performance was reported to be unsatisfactory for most genes due to predicted gene expression values not correlating well with the observed values (Li et al., 2018). Differences in prediction accuracy with PrediXcan between the YRI and CEU, however, were not directly compared. Gottlieb et al. investigated the performance of PrediXcan for a small subset of 116 genes that are in the warfarin-response pathway in European and African American samples where they concluded that PrediXcan performed poorly in African Americans (Gottlieb et al., 2017).

Here, we evaluate the predictive performance of PrediXcan both across and within continental populations using thousands of genes across the genome. Using the GEUVADIS transcriptome data and whole genome sequencing data from the 1000 Genomes Project (Lappalainen et al., 2013; Auton et al., 2015), we consider four closely related European ancestry populations and one African population. In our analysis, we test the null hypotheses of (1) no difference in prediction accuracy with PrediXcan across European and African continental populations; and (2) no difference in predictive performance among the four European derived populations. We obtain predicted gene expression levels using seven PrediXcan weight databases derived from whole blood and lymphoblastoid cell lines (LCL) transcriptome data for each individual. To evaluate differences in prediction accuracy among the populations, we use a linear mixed effects model framework where Pearson's correlation coefficients for observed and predicted gene expression levels are included as the outcome and the populations are included as categorical predictors. In addition, we evaluate the utility of whole-blood-based models when making predictions for LCL expression data. We find from our analyses that accuracy of PrediXcan for gene expression prediction not only differs between European and African continental populations, but also among closely related populations of European ancestry. Furthermore, prediction accuracy with PrediXcan is the lowest in Africans across all seven weight databases considered, which further illustrates the need to develop new predictive models using training data composed of individuals who have similar ancestry to the target sample for which gene expression is to be predicted (Mogil et al., 2018).

## 2. Materials and Methods

### 2.1. Datasets

We obtained gene expression data from the GEUVADIS Consortium and whole genome sequencing data from the 1000 Genomes Project. The gene expression data consisted of RNA sequencing on lymphoblastoid cell line (LCL) samples for 464 individuals from five populations. Of these, 445 subjects were in the 1000 Genomes Phase 3 dataset, including 358 subjects of European descent, and 87 subjects of African descent. European samples included: Utah residents with Northern and Western European ancestry (CEU, *n* = 89), British individuals in England and Scotland (GBR, *n* = 86), Finnish in Finland (FIN, *n* = 92), and Toscani in Italy (TSI, *n* = 91). African samples included individuals of African descent from Yoruba in Ibadan, Nigeria (YRI, *n* = 87). Gene expression measurements were available for 23,722 genes.

We used seven PrediXcan weight databases: DGN whole-blood (further referred to as DGN), GTEx v6 1KG whole blood, GTEx v6 1KG LCL, GTEx v6 HapMap whole blood, GTEx v6 HapMap LCL, GTEx v7 HapMap whole blood (GTEx WB), and GTEx v7 HapMap LCL (GTEx LCL). The databases were downloaded from http://predictdb.org/.

### 2.2. Filtering Procedure for Poorly Predicted Genes

Linear regression models were used to identify genes whose predicted values were not associated with the observed values at significance level of 0.05 in order to filter out genes that have poor prediction accuracy across all subjects. For each gene, we fit a linear regression model with observed gene expression as the outcome and predicted gene expression as the predictor of interest. A Wald test was used to assess significance of the coefficient for each gene in the linear model. Genes with corresponding *p*-values that were higher than a nominal significance level of 0.05 were identified and labeled as “poorly predicted.”

We then calculated Pearson's correlation coefficient, *r*, between observed and predicted expression values for every gene, in each population separately. A few genes had the same predicted gene expression levels across all subjects. Since we could not calculate the correlation if one of the variables was constant, we excluded those genes. Thus, for every gene considered there were five Pearson's correlation coefficients, one for each population. Note that we used *r* instead of the square of Pearson correlation, *r*^2^, in order to take directionality of correlation into account when assessing predictive performance. We found that using *r*^2^ as a measure of predictive accuracy can be misleading as there were genes for which predicted and observed expression values had a significant negative correlation.

It should be noted that we also performed an evaluation of the performance of PrediXcan without doing any filtering of genes in order to assess the impact on the analysis when poorly predicted genes are excluded, as discussed below.

### 2.3. Assessing Prediction Accuracy Differences Across Populations and Across Tissues

In the analyses described below to assess differences in prediction accuracy with PrediXcan across populations, two sets of genes were considered—all genes without any filtering and a subset of genes using the filtering process previously described.

We first compared prediction performance between the two continental groups—European and African. For each gene, we calculated two Pearson's correlation coefficients between observed and predicted gene expression levels—one based on all European samples and the other one based on the African samples. We then used a paired *t*-test to assess differences in mean prediction accuracy between the correlation coefficients for European samples vs correlation coefficients for African samples.

To assess differences in prediction accuracy across the five populations, we used a linear mixed effects model approach where we fit the following model:

(1) $$\begin{array}{l} r_{ij}=\beta_{0}+\gamma_{i}+\beta_{1}{𝕀}_{FIN,i}+\beta_{2}{𝕀}_{GBR,i}+\beta_{3}{𝕀}_{TSI,i}+\beta_{4}{𝕀}_{YRI,i}+\epsilon_{ij}, \end{array}$$

where *r*_*ij*_ is the correlation coefficient for gene *i* in population *j*; and 𝕀_*FIN,i*_, 𝕀_*GBR,i*_, 𝕀_*TSI,i*_, and 𝕀_*YRI,i*_ are indicator variables that are equal to 1 if the gene correlation was calculated on the population indicated in the subscript, and otherwise are equal to 0. Thus, we modeled population as a categorical predictor, with the CEU population as a reference. To account for variation between genes, we included a random intercept γ_*i*_ for each gene and we assumed that $\gamma_{i}\sim N\left(0,\sigma_{\gamma }^{2}\right)$. We also included an error term ϵ_*ij*_, such that $\epsilon_{ij}\sim N\left(0,\sigma^{2}\right)$. We used repeated measures ANOVA to test the null hypothesis of β_1_ = β_2_ = β_3_ = β_4_ = 0 for no difference in mean Pearson's correlation coefficients among the populations. A Wald test was used to assess significance of differences in mean Pearson's correlation coefficients between CEU, the reference population, and each of the other four populations.

We also ran a similar analysis where we excluded the CEU population due to potentially lower quality of the CEU cell lines, as reported in the literature (Çaliskan et al., 2014; Yuan et al., 2015). We fit a model identical to (1), excluding the CEU and using the FIN population as a reference:

(2) $$\begin{array}{l} r_{ij}=\beta_{0}+\gamma_{i}+\beta_{1}{𝕀}_{GBR,i}+\beta_{2}{𝕀}_{TSI,i}+\beta_{3}{𝕀}_{YRI,i}+\epsilon_{ij}, \end{array}$$

where the notation is the same as above.

Additionally, we tested for differences in prediction accuracy across the four European populations. For this analysis, we included only individuals of European ancestry and fit the following linear mixed effects model:

(3) $$\begin{array}{l} r_{ij}=\beta_{0}+\gamma_{i}+\beta_{1}{𝕀}_{FIN,i}+\beta_{2}{𝕀}_{GBR,i}+\beta_{3}{𝕀}_{TSI,i}+\epsilon_{ij}, \end{array}$$

where CEU is included as the reference population in the model. As in the previously described analyses, a repeated measures ANOVA was used to test for differences in prediction accuracy across the four European populations.

To evaluate how the PrediXcan performance with whole-blood (WB) databases differed from LCL databases, we restricted the set of genes to only those that were present in both the WB and LCL databases. First, we presented scatter plots of correlation coefficients comparing WB and LCL databases in the five populations separately. Then we recalculated Pearson's correlation coefficients between observed and predicted expression values with all the five populations combined but separately for every database, i.e., as a result, we had two correlation coefficients per gene, one that corresponded to a GTEx WB database and one to a GTEx LCL database. We compared each pair of GTEx WB and GTEx LCL databases using a paired *t*-test between LCL-based correlation coefficients and WB-based correlation coefficients. All the statistical analyses described above were performed in R version 3.3.3 (R Core Team, 2014). All plots were generated with ggplot2 (Wickham, 2016).

## 3. Results

### 3.1. Overview of PrediXcan Weight Databases

In Table 1, we summarize the main features of the PrediXcan weight databases that we used in the analyses. Compared to DGN database, GTEx databases have fewer gene models and smaller training sample sizes. HapMap and 1KG-based models differ in the number of variants used for training: GTEx Hapmap models were trained on the HapMap genotyping data while GTEx 1KG were trained on the 1000 Genomes sequencing data, so the latter utilize more variants when predicting expression. While GTEx LCL databases are based on relatively small training sets, they are derived from the same tissue as the GEUVADIS RNA-seq data we analyzed. Lastly, DGN and GTEx v7 sets of weights were trained only on Europeans samples, while GTEx v6 databases had a small fraction of non-Europeans.

**Table 1:** Summary of PrediXcan databases used in analyses.

**PrediXcan database**	**Training set size**	**Number of models**	**Number of SNPs used**
DGN whole blood	922	13,171	249,696
GTEx v6 1KG whole blood	338	6,759	185,786
GTEx v6 1KG LCL	114	3,759	125,045
GTEx v6 HapMap whole blood	338	6,588	136,941
GTEx v6 HapMap LCL	114	3,441	91,237
GTEx v7 HapMap whole blood	315	6,297	140,931
GTEx v7 HapMap LCL	96	3,045	88,143

To avoid repetition, results using the DGN, GTEx v7 WB, and GTEx v7 LCL databases are included in the main text, while the results for the other four databases are provided in the Supplementary Material.

### 3.2. PrediXcan Prediction Accuracy Differs Across Diverse Populations

Using DGN, GTEx WB, and GTEx LCL models and sequence data, gene expression was predicted for 10,387, 5,432, and 2,777 genes, respectively (see Table 2). The number of genes with available predictions varied by population, where the four European populations had a similar number of gene predictions while the counts for YRI were slightly lower. We excluded 33 genes, 13 genes, and 10 genes from DGN, GTEx WB, and GTEx LCL, respectively, due to there being no variation in predicted gene expression values for at least one of the populations. For the remaining genes, we identified those that had poor prediction accuracy based on associations between observed and predicted values, as described in section Materials and Methods on filtering poorly predicted genes. From the genes predicted with the DGN database, two-thirds were labeled by this criterion as “poorly predicted,” while slightly less than a half were labeled as such from gene sets predicted using the GTEx databases. As previously mentioned, we also considered the performance of PrediXcan without doing any filtering of the genes. For every weight database, we had two sets of genes—before and after filtering—where the latter set is a much smaller subset of the former. Both versions were used and evaluated in downstream analyses.

**Table 2:** Number of genes for which Pearson correlation coefficients are available by population and by PrediXcan weight database.

**PrediXcan database**	**DGN**	**GTEx v7 WB**	**GTEx v7 LCL**
Genes with observed and	10,387	5,432	2,777
predicted expression values			
By population:			
CEU	10,385	5,432	2,777
FIN	10,385	5,432	2,777
GBR	10,385	5,432	2,777
TSI	10,385	5,432	2,776
YRI	10,354	5,419	2,767
Genes before filtering	10,354	5,419	2,767
Genes after filtering	3,493	2,288	1,699

We first evaluated performance of PrediXcan for the two continental populations, European and African. We compared Pearson's correlation of predicted and observed gene expression values for the combined sample consisting of all individuals from the four European-ancestry populations to Pearson's correlation calculated for the YRI African population sample. As only two groups were being compared in this analysis, a paired *t*-test was used to assess differences in prediction accuracy, where the pairing was based on the gene. With or without the filtering of genes, we find the mean difference in gene correlation coefficients between the European and African samples to be highly significantly different from zero, regardless of the weight database used (all *p*-values < 2.2 × 10^−16^), with the African population having lower prediction accuracy than the European samples.

**Figure 1:**
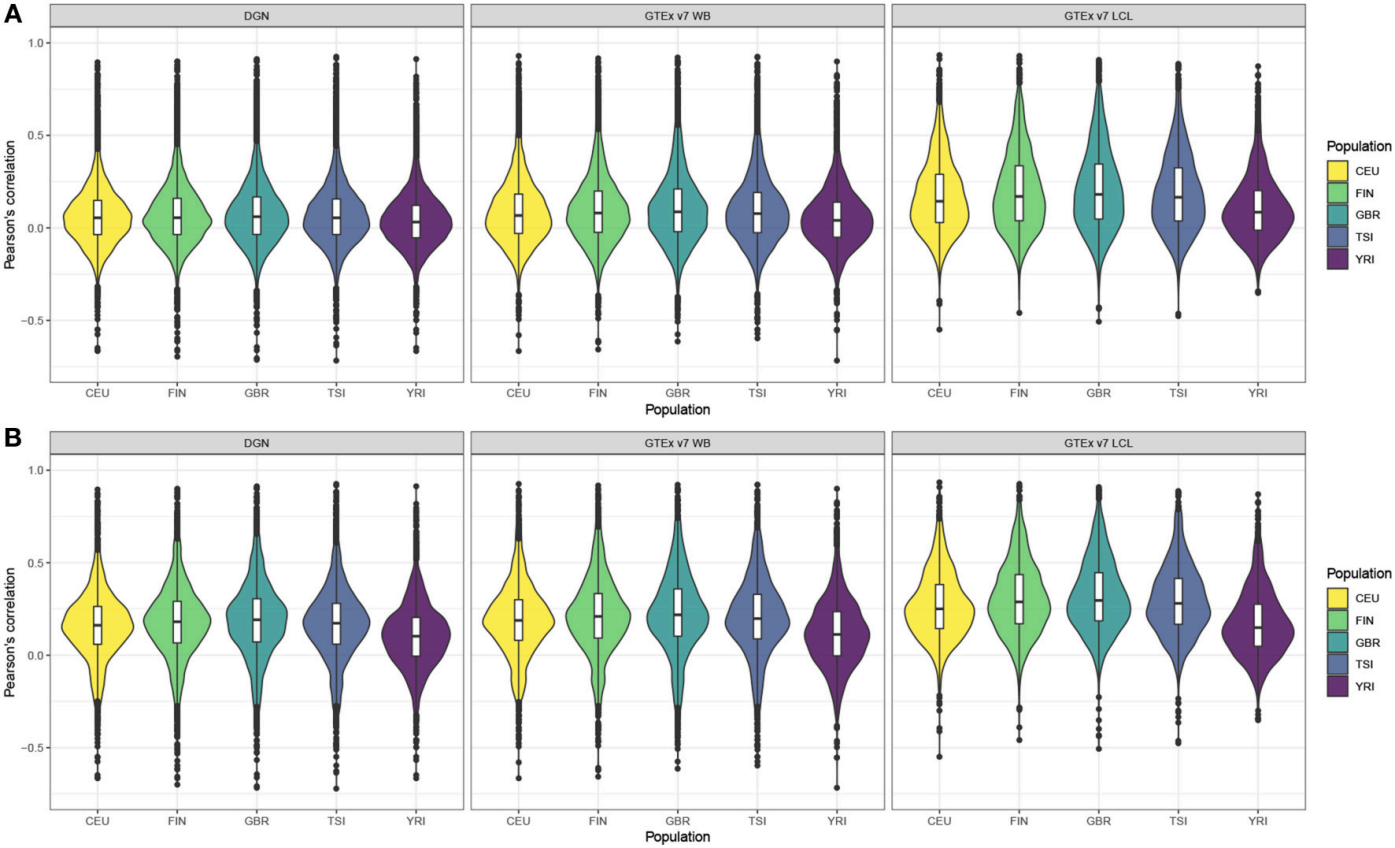
Violin plots of gene expression correlation coefficients by five populations using DGN, GTEx v7 WB, and GTEx v7 LCL weight databases; **(A)** before and **(B)** after filtering out poorly predicted genes.

Next, we computed gene correlation coefficients, separately in each of the five populations. Violin plots display the correlation coefficients by population across genes before and after filtering (see Figures 1A,B, respectively). Figure 1A shows correlation coefficients for the genes before any filtering was done and we observe that LCL-derived models perform better than WB-derived: i.e., DGN and GTEx v7 WB correlation distributions are centered at values close to 0, whereas GTEx LCL correlation distributions are centered at higher values, especially for the four European populations. We also note that prediction accuracy is slightly lower for the African populations than for any of the European populations across the three weight databases. This trend is even more obvious after the filtering process. As we can see in Figure 1B, the overall performance accuracy improved after filtering in all the populations, as expected. However, the difference in prediction performance in Europeans vs. Africans is even more apparent. The four European populations have similar prediction accuracy, whereas it is lower for the African population. Similarly to panel A, LCL-derived prediction models perform better than WB-derived in filtered genes in Figure 1B.

Afterwards, we binned the genes into six categories based on the gene correlation coefficients (see Table 3). The majority of genes have very poor prediction accuracy—of the genes predicted with whole-blood databases, a third have negative correlations and a half have correlations between 0 and 0.2. Of the genes predicted with LCL, a fifth have negative correlations and over a third have correlations between 0 and 0.2. The distribution of gene correlation coefficients is fairly similar across the four European populations, although predictive accuracy seems worse in CEU compared to FIN, GBR, and TSI. The predictive accuracy is the lowest in the African sample. Across all populations, only a small number of genes were predicted with high accuracy (with *r* > 0.6). Furthermore, all European populations have a greater number of well-predicted genes than the African population, regardless of the weight database used.

**Table 3:** Gene counts per population, per database, per correlation category for the five populations using DGN, GTEx WB, and GTEx LCL weight databases.

	**Unfiltered**					**Filtered**				
	**CEU**	**FIN**	**GBR**	**TSI**	**YRI**	**CEU**	**FIN**	**GBR**	**TSI**	**YRI**
**DGN DATABASE**										
*r* < 0	3,583	3,491	3,480	3,587	4,156	561	547	554	585	911
0 < *r* < 0.2	5,107	4,976	4,812	4,954	5,001	1,533	1,379	1,258	1,409	1,674
0.2 < *r* < 0.4	1,359	1,480	1,589	1,434	1,016	1,097	1,162	1,209	1,121	728
0.4 < *r* < 0.6	239	302	354	290	147	236	300	353	289	146
0.6 < *r* < 0.8	56	93	105	75	31	56	93	105	75	31
0.8 < *r* < 1	10	12	14	14	3	10	12	14	14	3
**GTEx v7 WB DATABASE**										
*r* < 0	1,756	1,621	1,622	1,684	2,101	336	309	314	335	590
0 < *r* < 0.2	2,471	2,450	2,366	2,456	2,491	877	786	732	820	993
0.2 < *r* < 0.4	902	958	981	901	668	788	804	793	758	546
0.4 < *r* < 0.6	210	282	329	278	117	207	281	328	275	117
0.6 < *r* < 0.8	69	93	100	85	38	69	93	100	85	38
0.8 < *r* < 1	11	15	21	15	4	11	15	21	15	4
**GTEx v7 LCL DATABASE**										
*r* < 0	546	488	484	509	774	80	69	55	69	274
0 < *r* < 0.2	1,119	1,031	996	1,050	1,296	560	443	426	477	777
0.2 < *r* < 0.4	718	742	761	736	510	675	681	692	681	461
0.4 < *r* < 0.6	293	361	369	360	145	293	361	369	360	145
0.6 < *r* < 0.8	80	126	137	96	38	80	126	137	96	38
0.8 < *r* < 1	11	19	20	16	4	11	19	20	16	4

Next, we assessed the association between the prediction accuracy (as gene correlation coefficients) and population category via repeated measures ANOVA and linear mixed models using both sets of genes, all and filtered. The results for unfiltered and filtered genes were comparable and led to equivalent conclusions. Based on the repeated measures ANOVA, we find that prediction accuracy differs across populations for filtered and unfiltered sets of genes, regardless of the weight database used (*p*-values for all databases were < 2.2 × 10^−16^). Below, we focus our attention on filtered genes and present the parameter estimates and their 95% confidence intervals calculated using model-based standard errors for the model 1 in Table 4. From the linear mixed model 1, we find that the prediction accuracy is significantly higher in FIN, GBR, and TSI and significantly lower in YRI, compared to CEU. This suggests that predictive performance varies not only among distant populations, but also among closely related populations. When we performed the analysis on a full set of genes, without any filtering, regression coefficients were slightly attenuated toward zero; however, the conclusions from hypothesis testing remained the same.

**Table 4:** Results from linear mixed models for population category (with CEU as a reference) and change in gene correlation coefficient among filtered genes.

	**DGN**			**GTEx v7 WB**			**GTEx v7 LCL**		
	**Estimate**	**95% CI**	***p*-value**	**Estimate**	**95% CI**	***p*-value**	**Estimate**	**95% CI**	***p*-value**
FIN	0.019	(0.014, 0.025)	1.3 × 10^−11^	0.021	(0.015, 0.028)	1.3 × 10^−9^	0.038	(0.030, 0.046)	< 10^−16^
GBR	0.029	(0.023, 0.034)	< 10^−16^	0.032	(0.025, 0.039)	< 10^−16^	0.051	(0.043, 0.059)	< 10^−16^
TSI	0.010	(0.004, 0.016)	3.9 × 10^−4^	0.013	(0.007, 0.020)	4.6 × 10^−5^	0.027	(0.019, 0.035)	2.9 × 10^−11^
YRI	−0.054	(−0.059, −0.048)	< 10^−16^	−0.070	(−0.077, −0.063)	< 10^−16^	−0.097	(−0.105 −0.089)	< 10^−16^

We repeated the analysis described above, this time excluding the CEU population. We present the parameter estimates and the corresponding 95% confidence intervals in Table 5. From the repeated measures ANOVA, we find that prediction accuracy differs across the four populations (*p*-values for all databases were < 2.2 × 10^−16^). Moreover, based on the coefficients and the corresponding *p*-values from the linear mixed model 2, we estimate the prediction accuracy to be significantly higher in GBR and significantly lower in TSI and YRI, compared to the FIN population (see corresponding *p*-values in Table 5). This difference in prediction accuracy is the greatest between YRI and FIN when GTEx v7 LCL weight database was used. Like in the analysis above, we notice that predictive performance differs across populations, including European populations.

**Table 5:** Results from linear mixed models for population category (excluding CEU, with FIN as a reference) and change in gene correlation coefficient among filtered genes.

	**DGN**			**GTEx v7 WB**			**GTEx v7 LCL**		
	**Estimate**	**95% CI**	***p*-value**	**Estimate**	**95% CI**	***p*-value**	**Estimate**	**95% CI**	***p*-value**
GBR	0.010	(0.004, 0.015)	9.2 × 10^−4^	0.011	(0.004, 0.018)	3.1 × 10^−3^	0.013	(0.005, 0.021)	2.0 × × 10^−3^
TSI	−0.009	(−0.015, −0.003)	1.8 × 10^−3^	−0.008	(−0.015, −0.001)	2.8 × 10^−2^	−0.011	(−0.019, −0.003)	8.9 × 10^−3^
YRI	−0.073	(−0.079, −0.067)	< 10^−16^	−0.091	(−0.098, −0.084)	< 10^−16^	−0.134	(−0.143, −0.126)	< 10^−16^

Finally, we evaluated PrediXcan prediction accuracy on a subset of subjects with European ancestry. Based on the repeated measures ANOVA test, prediction performance differs across the four European populations in genes before and after filtering, regardless of the weight database used (*p*-values for all databases were < 2.2 × 10^−16^). Because of potentially biased expression patterns of the CEU due to the previously mentioned age of these cell lines, we conducted an analysis where we omitted the CEU population and compared prediction accuracy among the other three European populations. The results were comparable to the analysis of the European populations that included CEU. With a repeated measures ANOVA, we find highly significant differences in prediction accuracy among the FIN, GBR, and TSI populations, with *p*-values less than 10^−6^ across all weight databases with or without filtering of poorly predicted genes.

### 3.3. PrediXcan Prediction Accuracy Differs Between Tissues

As can be seen in the violin plots in Figure 1, both databases based on whole blood perform similarly, and LCL-based database displays improved prediction accuracy. In order to compare pairwise gene correlations, we restricted our analyses to the 1,595 genes common for both GTEx v7 WB and GTEx v7 LCL.

**Figure 2:**
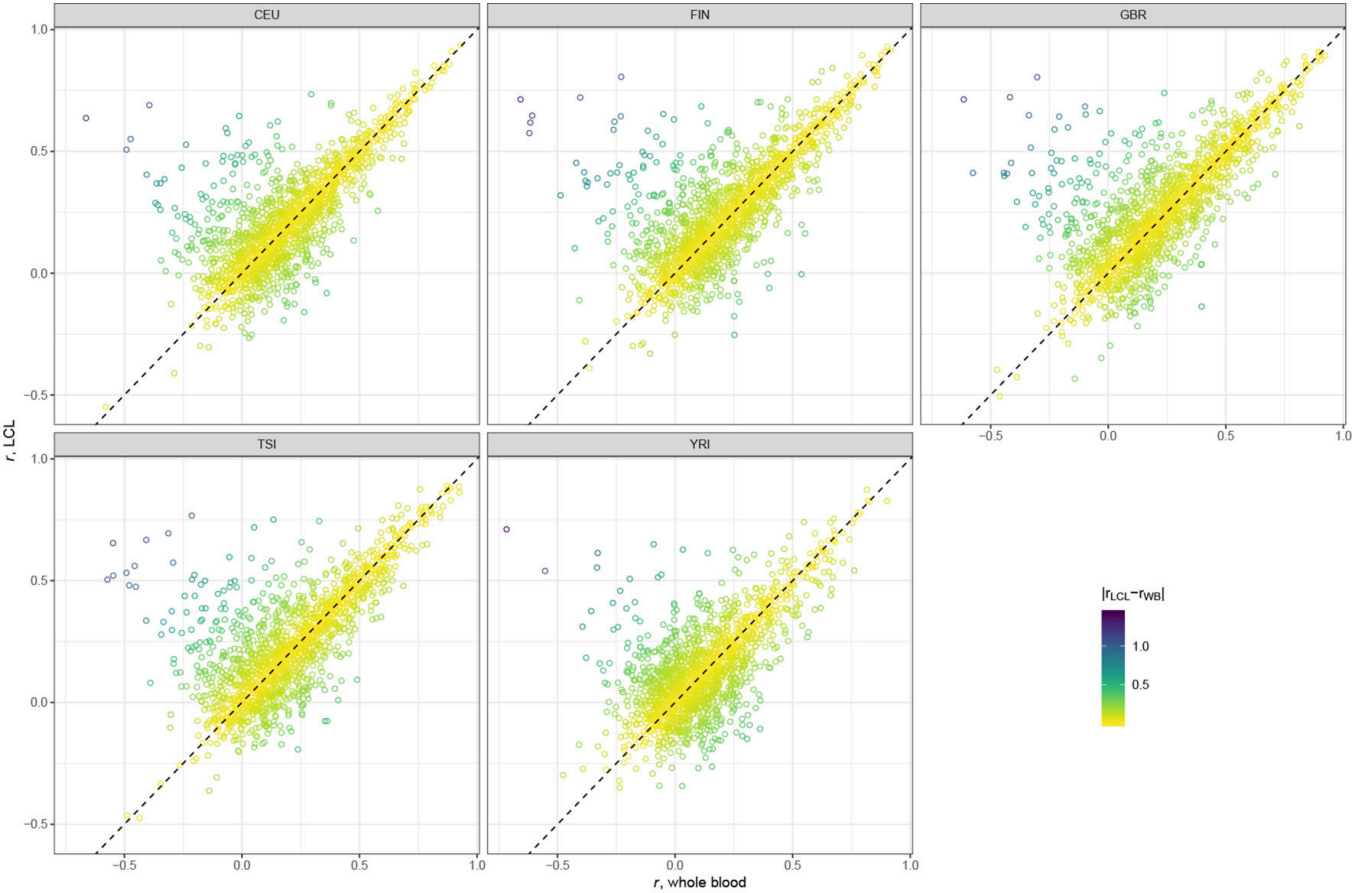
Scatter plots comparing gene correlation coefficients by population using GTEx v7 LCL vs. GTEx v7 WB databases.

Scatter plots presented in Figure 2 suggest that the majority of genes have very similar correlation coefficients when using WB and LCL databases across all populations. However, we see more genes in the upper left corner, above the dotted line, indicating that using the LCL database results in more genes with better prediction accuracy. This result is not surprising since the expression data we used were derived from LCL. The results of the paired *t*-test are consistent with the visual examination of the data: the mean difference between gene correlations based on the GTEx v7 LCL models and based on the GTEx v7 WB models is 0.03 (*p*-value < 2.2 × 10^−16^), with predictions based on the LCL model having higher performance.

## 4. Discussion

In this work, we evaluated the performance of PrediXcan and compared the prediction accuracy of the method across five geographically diverse populations from two continents for seven weight databases. Models from all weight databases considered were trained on subjects primarily of European ancestry; three of the databases were derived from LCL and the remaining four from whole blood. As a measure of prediction accuracy, we computed correlation coefficients for each gene in all populations and used both paired *t*-tests and linear mixed effects models to assess evidence of significant differences in prediction performance across populations. We also investigated whether whole blood models are appropriate for predicting gene expression levels in LCL.

We find highly significant differences in prediction accuracy with PrediXcan in the European ancestry populations as compared to the YRI African population, with the prediction accuracy being lower in YRI. The lower accuracy with PrediXcan in the African population is expected since the PrediXcan models were largely trained using European ancestry samples, and this result is consistent with recent works showing that prediction accuracy is expected to be higher when the training and testing cohorts are of similar ancestry (Gottlieb et al., 2017; Li et al., 2018; Mogil et al., 2018). Surprisingly, we also find highly significant differences in prediction accuracy with PrediXcan among the closely related European ancestry populations, with the Finnish, British, and Italian populations having significantly higher prediction accuracy than the CEU. These results are consistent across all seven PrediXcan weight databases we considered. Lastly, we also find that LCL-trained models outperformed whole-blood-trained models across populations, although the prediction accuracy was similar for many of the genes.

Among the European populations, we find that prediction accuracy for the CEU population was the lowest. LCLs are derived from B cells found in whole blood, and they provide a continuous supply of genetic material for GWAS and gene expression studies. However, they do undergo a transformation to become immortal that can change their biology and they do not have the same properties as native tissue (Kelly et al., 2017). Storage conditions, freeze-thaw cycles, and maturity of cell lines can also affect gene expression patterns (Çaliskan et al., 2014; Yuan et al., 2015). The CEU cell lines were collected much earlier than the other cell lines and LCL age can have a confounding effect and bias downstream analyses (Yuan et al., 2015). This factor could have contributed to the differences in prediction accuracy among European populations. We did, however, perform a sensitivity analysis that excluded the CEU population, and there were highly significant differences in prediction accuracy with PrediXcan among the FIN, GBR, and TSI populations, as well as between these three combined European populations and the YRI African population, with the YRI having the lowest accuracy.

Overall, PrediXcan accurately predicted gene expression for some genes; however, the majority of genes had very poor correlation between measured and predicted expression levels. For almost half the genes, for example, the correlation was negative. There are some important caveats and limitations to point out with the PrediXcan method. First, the prediction models of PrediXcan are based on common *cis*-variants and they do not take rare *cis*- and *trans*-regulatory elements into account. Common *cis*-eQTLs only account for 9–12% of genetic variance in gene expression, according to a large twin study (Grundberg et al., 2012). Another recent study demonstrates that *trans*-acting variants largely contribute to gene expression variation, with estimates of genetic variance in expression due to *trans*-acting variation ranging from 60 to 90% (Liu et al., 2018). However, individual effects of each *trans*-variant are very weak and difficult to map because they require well-powered studies.

We conclude this paper by highlighting that the lack of genomic data from diverse populations limits the ability to effectively interpret and translate genomic results into clinical applications for individuals from diverse populations, and particularly non-European ancestry populations. The results presented in this paper illustrate that gene expression prediction models are, in general, not transferable across diverse populations from different continents, and further corroborate the importance of including more ancestrally diverse individuals in medical genomics to ensure that everyone gets the benefits of precision medicine and to avoid further exacerbating healthcare inequality (Oh et al., 2015, 2016; Manrai et al., 2016). We also demonstrate that there can be differences in prediction accuracy among closely related European populations, suggesting that prediction models that take into account fine-scale ancestry differences among individuals may be important for improved prediction of gene expression from genetic data. Lastly, our study had only modest sample sizes and evaluated gene expression prediction accuracy with PrediXcan in European and African populations. Future transcriptomic studies with much larger samples sizes are needed for the development of improved gene expression prediction models for multi-ethnic populations, including admixed populations such as African Americans and Hispanic/Latino populations, who have recent ancestry derived from multiple continents.

## Data Availability

GEUVADIS expression data is available at Array Express (E-MTAB-264 and E-GEUV-1) at https://www.ebi.ac.uk/arrayexpress/experiments/ and 1000 Genomes project genotype data is available at http://www.internationalgenome.org/.

## Author Contributions

AM and TT conceived the idea, designed the analysis, interpreted the results, and wrote the paper. AM ran the analysis.

### Conflict of Interest Statement

The authors declare that the research was conducted in the absence of any commercial or financial relationships that could be construed as a potential conflict of interest.
