# Supplementary material for: Accuracy of Gene Expression Prediction From Genotype Data With PrediXcan Varies Across and Within Continental Populations
Source: Front Genet. 2019 Apr 3;10:261. doi: 10.3389/fgene.2019.00261 (PMC6456650; doi:10.3389/fgene.2019.00261)
Supplement: Supplementary file 1 [file Data_Sheet_1.pdf]

Supplementary Material: Accuracy of gene expression prediction from genotype data with PrediXcan varies across and within continental populations

1 SUPPLEMENTARY TABLES AND FIGURES

We present the results for the four PrediXcan weight databases omitted from the main text. The databases are GTEx v6 1KG whole blood (GTEx v6 1KG WB), GTEx v6 1KG LCL (GTEx v6 1KG LCL), GTEx v6 HapMap whole blood (GTEx v6 HM WB), and GTEx v6 HapMap LCL (GTEx v6 HM LCL).

Table S1. Number of genes for which Pearson correlation coefficients are available by population and by GTEx v6 database.

PrediXcan database	1KG WB	1KG LCL	HM WB	HM LCL
Genes with observed and predicted expression values	6,179	3,662	6,039	3,363
By population:				
CEU	6,136	3,636	6,017	3,361
FIN	6,132	3,637	6,020	3,361
GBR	6,133	3,637	6,018	3,361
TSI	6,141	3,637	6,025	3,361
YRI	6,039	3,612	5,997	3,351
Genes before filtering	6,010	3,604	5,978	3,350
Genes after filtering	2,198	1,889	2,207	1,847

Table S2. Binned gene correlation coefficients for the five populations using GTEx v6 weight databases.

	Unfiltered					Filtered				
	CEU	FIN	GBR	TSI	YRI	CEU	FIN	GBR	TSI	YRI
	GTEx v6 1KG WB database									
$r < 0$	2,094	2,031	1,993	2,051	2,285	347	313	329	334	492
$0 < r < 0.2$	2,804	2,747	2,673	2,770	2,826	870	809	730	804	976
$0.2 < r < 0.4$	856	892	961	867	708	727	737	757	739	540
$0.4 < r < 0.6$	183	249	280	240	147	181	248	279	239	146
$0.6 < r < 0.8$	64	82	88	69	39	64	82	88	69	39
$0.8 < r < 1$	9	9	15	13	5	9	9	15	13	5
	GTEx v6 1KG LCL database									
$r < 0$	841	806	799	804	1030	97	99	86	100	236
$0 < r < 0.2$	1,570	1,492	1,460	1,544	1,666	673	570	551	612	817
$0.2 < r < 0.4$	849	841	833	829	676	775	755	740	750	604
$0.4 < r < 0.6$	267	339	376	320	178	267	339	376	320	178
$0.6 < r < 0.8$	69	112	117	89	48	69	112	117	89	48
$0.8 < r < 1$	8	14	19	18	6	8	14	19	18	6
	GTEx v6 HapMap WB database									
$r < 0$	2,092	2,052	1,986	2,065	2,246	350	331	329	368	504
$0 < r < 0.2$	2,770	2,705	2,679	2,760	2,883	885	804	763	830	1,004
$0.2 < r < 0.4$	879	892	942	846	685	736	743	745	703	535
$0.4 < r < 0.6$	176	248	279	229	128	175	248	278	228	128
$0.6 < r < 0.8$	55	72	79	68	33	55	72	79	68	33
$0.8 < r < 1$	6	9	13	10	3	6	9	13	10	3
	GTEx v6 HapMap LCL database									
$r < 0$	750	691	676	688	947	87	77	89	81	256
$0 < r < 0.2$	1,471	1,405	1,365	1,434	1,568	689	593	536	601	820
$0.2 < r < 0.4$	810	797	847	834	628	752	720	760	771	564
$0.4 < r < 0.6$	257	350	328	299	164	257	350	328	299	164
$0.6 < r < 0.8$	53	93	119	78	39	53	93	119	78	39
$0.8 < r < 1$	9	14	15	17	4	9	14	15	17	4

Table S3. Results from linear mixed models for population category (with CEU as a reference) and change in gene correlation coefficient among filtered genes.

Regression	GTEx v6 1KG WB		GTEx v6 1KG LCL		GTEx v6 HM WB		GTEx v6 HM LCL	
parameter	Estimate	95% CI	Estimate	95% CI	Estimate	95% CI		
FIN	0.025	(0.018, 0.032)	0.031	(0.024, 0.039)	0.020	(0.013, 0.027)	0.034	(0.026, 0.041)
GBR	0.031	(0.023, 0.038)	0.043	(0.035, 0.050)	0.028	(0.021, 0.035)	0.044	(0.036, 0.051)
TSI	0.015	(0.008, 0.022)	0.023	(0.016, 0.031)	0.011	(0.004, 0.018)	0.027	(0.019, 0.034)
YRI	-0.040	(-0.047, -0.033)	-0.061	(-0.069, -0.054)	-0.047	(-0.054, -0.040)	-0.064	(-0.071, -0.056)

Table S4. Results from linear mixed models for population category (excluding CEU, with FIN as a reference) and change in gene correlation coefficient among filtered genes.

Regression	GTEx v6 1KG WB		GTEx v6 1KG LCL		GTEx v6 HM WB		GTEx v6 HM LCL	
parameter	Estimate	95% CI	Estimate	95% CI	Estimate	95% CI		
GBR	0.006	(-0.001, 0.013)	0.012	(0.004, 0.019)	0.008	(0, 0.015)	0.010	(0.002, 0.018)
TSI	-0.009	(-0.017, -0.002)	-0.008	(-0.016, 0)	-0.010	(-0.017, -0.002)	-0.007	(-0.015, 0)
YRI	-0.064	(-0.072, -0.057)	-0.092	(-0.100, -0.085)	-0.067	(-0.075, -0.060)	-0.099	(-0.107, -0.091)

1.1 Figures

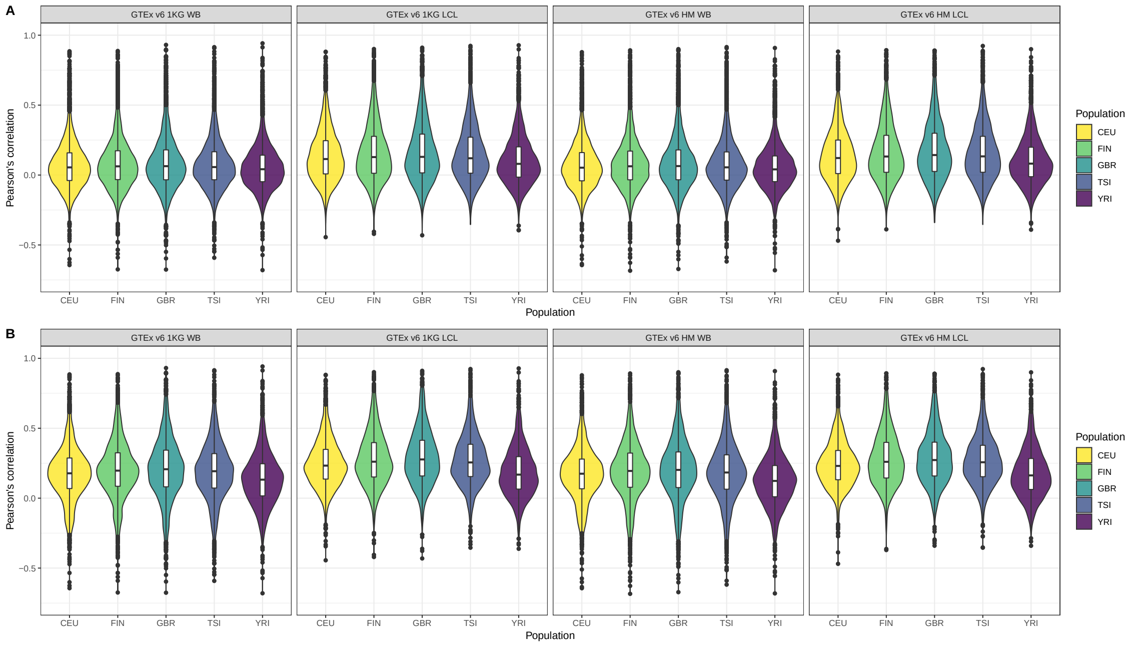

Figure S1. Violin plots of gene expression correlation coefficients by five populations using GTEx weight databases; **(A)** before and **(B)** after filtering out poorly predicted genes.

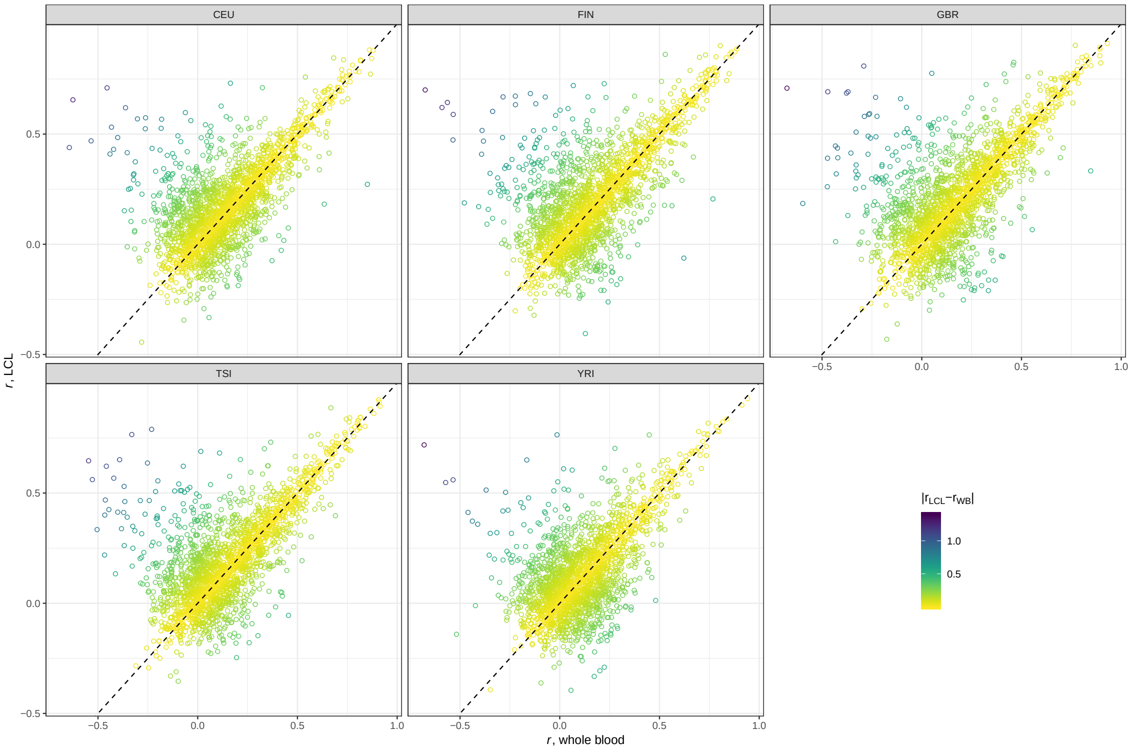

Figure S2. Scatter plots comparing gene correlation coefficients by population using GTEx v6 1KG LCL vs GTEx v6 1KG WB databases.

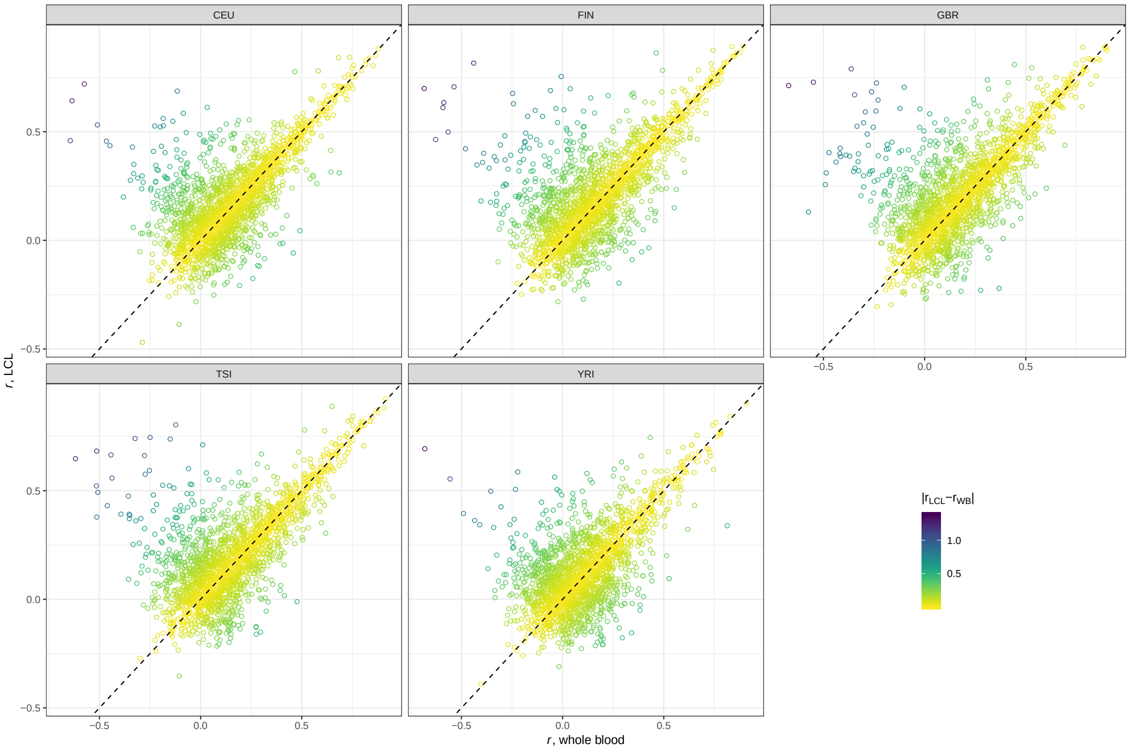

Figure S3. Scatter plots comparing gene correlation coefficients by population using GTEx v6 HapMap LCL vs GTEx v6 HapMap WB databases.
